# Non-invasive Differentiation of Kidney Stone Types using X-ray Dark-Field Radiography

**DOI:** 10.1038/srep09527

**Published:** 2015-04-15

**Authors:** Kai Scherer, Eva Braig, Konstantin Willer, Marian Willner, Alexander A. Fingerle, Michael Chabior, Julia Herzen, Matthias Eiber, Bernhard Haller, Michael Straub, Heike Schneider, Ernst J. Rummeny, Peter B. Noël, Franz Pfeiffer

**Affiliations:** 1 Lehrstuhl für Biomedizinische Physik, Physik-Department & Institut für Medizintechnik, Technische Universität München, Garching, Germany; 2 Department of Radiology, Technische Universität München, Munich, Germany; 3 Department of Medical Statistics and Epidemiology, Technische Universität München, Munich, Germany; 4 Department of Urology, Technische Universität München, Munich, Germany; 5 Department of Clinical Chemistry and Pathobiochemistry, Technische Universität München, Munich, Germany

## Abstract

Treatment of renal calculi is highly dependent on the chemical composition of the stone in question, which is difficult to determine using standard imaging techniques. The objective of this study is to evaluate the potential of scatter-sensitive X-ray dark-field radiography to differentiate between the most common types of kidney stones in clinical practice. Here, we examine the absorption-to-scattering ratio of 118 extracted kidney stones with a laboratory Talbot-Lau Interferometer. Depending on their chemical composition, microscopic growth structure and morphology the various types of kidney stones show strongly varying, partially opposite contrasts in absorption and dark-field imaging. By assessing the microscopic calculi morphology with high resolution micro-computed tomography measurements, we illustrate the dependence of dark-field signal strength on the respective stone type. Finally, we utilize X-ray dark-field radiography as a non-invasive, highly sensitive (100%) and specific (97%) tool for the differentiation of calcium oxalate, uric acid and mixed types of stones, while additionally improving the detectability of radio-lucent calculi. We prove clinical feasibility of the here proposed method by accurately classifying renal stones, embedded within a fresh pig kidney, using dose-compatible measurements and a quick and simple visual inspection.

The correct identification of the different renal calculi commonly found in the human body is of essential importance for the correct diagnosis, prognosis and therapy of many common diseases of the genitourinary system. For example, while urinary acid stones can occur in any healthy subject, struvite stones indicate an infection within the patient. In therapy, lithotripsy can be successfully administered for the uric acid type of kidney stones, while other types of calculi are more resistant to this type of therapy^1^,^2^. Nevertheless, while standard imaging methods like computed tomography and sonography are helpful in localizing calculi in the body, they only yield modest results in the correct identification of the stone type^3^,^4^. An examination of the patient's urine, or a removal of exemplary stones followed by a histological work-up is required in most cases. Currently, dual energy CT is evaluated with some success^5^.

Recent developments in phase-sensitive X-ray imaging^6^,^7^,^8^,^9^ have broadened the horizon of X-ray image contrast generation and are currently being evaluated for clinical application in a variety of diagnostic fields^10^. Among these, X-ray dark-field imaging^11^ attracted particular interest, being sensitive towards structural changes in the micro-morphology of tissue, as for instance associated with pathological processes of breast and lung tissue^12^,^13^,^14^. In contrast to absorption-based imaging, which solely relies on the reduction of beam intensity when introducing a specimen, dark-field contrast is generated by diffuse angular deflections of the X-ray wave-front when being scattered at inherent sub-structures. By resolving the scatter associated reduction of a phase-grating induced interference pattern, the dark-field signal strength can be quantified, as illustrated in Fig. 1 (for a detailed description of the technique, see Ref. 8). The dark-field signal has been shown to be highly dependent not only on the chemical composition of the imaged sample, but decisively also on the sample's morphological structure on the micrometer scale^15^,^16^, well below the resolution limit of commonly used imaging detectors.

The idea underlying our present work is to try to discriminate uric acid, calcium oxalate and mixed types of stones from each other within a radiographic imaging mode, on the ground of the complementarity of their absorption and dark-field contrasts, which is based on differences between their morphological and chemical compositions. While the absorption and dark-field images will be obtained from a lab-based radiography setup, the micro-morphological information (which are used to illustrate the generation of dark-field signal strength) will be assessed using highly resolving micro-CT.

## Results

### Analytical description

Formally, the measured projection value in absorption contrast 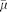 can be written as
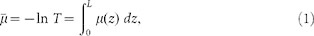
where the transmission *T* = *I*/*I*_0_ is obtained from the measured intensity *I*, relative to a reference intensity *I*_0_ measured without the kidney stone, *μ* being the absorption per unit length and *L* the stone thickness. As shown by Bech et al.^17^, under the simplified assumption of ideally random scattering, the dark-field signal in projection mode can similarly be written as
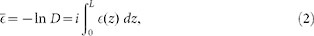
where the dark-field signal *D* = *V*/*V*_0_ can be obtained from the interferometric visibilities *V* and *V*_0_ with and without stone, respectively, 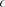 being the linear diffusion coefficient, quantifying the scattering per unit length, and i being a setup-specific constant.

To account for the problem of overlaying structures in projection mode, we can formally describe the kidney stone as consisting of a perfectly homogeneous material along each projection path, and assign to this hypothetical material an “effective” absorption and scattering coefficient *μ*_eff_ and 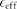, respectively,
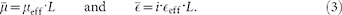


The effective coefficients *μ*_eff_ and 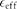 are thus defined as a weighted average of the contribution of the absorption and scattering coefficients along each projection path
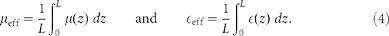


Interpreted in this way, the ratio of the projection values
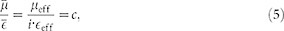
can be seen to be independent of the total kidney stone thickness *L*. Thus, in this approximation, we assume that there is a linear relationship between the measured 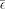 (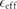) and 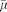 (*μ*_eff_) values, and that the slope *c* relating the two parameters is constant and characteristic for each kidney stone type. The simultaneous measurement of absorption and scattering thus allows the cancellation of the thickness dependence in projection mode, as well as the identification and classification of different kidney stones by using the obtained slope *c* as a binary classifier.

### Absorption characteristics of renal calculi

The effective absorption coefficient *μ*_eff_ is proportional to 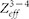, whereas the effective atomic number *Z_eff_* of the composite is mostly determined by the heaviest element in the kidney stone^18^. Thus, with respect to absorption, two classes of kidney stones can immediately be differentiated: the uric acid type of stones on the one hand, and the calcified stones (the oxalate, brushite and apatite/dahllite) on the other hand. While the heavy calcium ion in the calcified stones leads to a strong absorption signal (*Z_eff_* ≈ 14–16, large *μ*_eff_), the uric acid stones contain only low Z elements like carbon, nitrogen and oxygen which implies a small absorption signal (*Z_eff_* ≈ 7, small *μ*_eff_), respectively. Struvite and cystine have intermediate atomic numbers (*Z_eff_* ≈ 10–12). Nevertheless, those chemicals mostly occur only in combination with other crystallite phases and thus usually fall into the mixed stone category, with effective absorption coefficients *μ*_eff_ ranging between the uric acid and calcium oxalate class.

### Scattering characteristics of renal calculi

**Figure 2:**
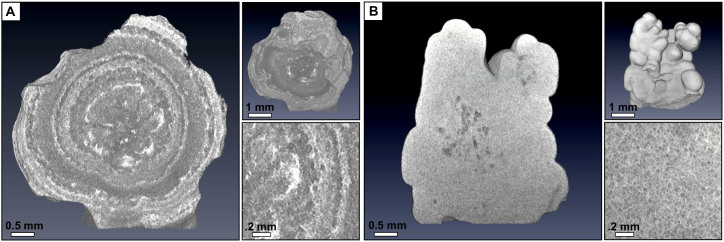
Comparison of the uric acid and calcium oxalate stone micro-morphology. (A) The volumetric micro-CT rendering of an uric acid-type kidney stone reveals a highly concentric growth structure, accompanied by a particular rough stone surface. The zoom-in of the tomographic slice shows high textural and optical irregularity induced by the multi-shell structure. (B) The volumetric micro-CT rendering of the calcium oxalate (90% mono-hydrate and 10% di-hydrate) stone, showing a strongly homogeneous micro-structure with a smooth stone exterior. The zoom-in of the tomographic slice reveals fine, regularly distributed crystallite cores on the micrometer scale. The non-uniformity in optical density and structure in the case of the uricite stone compared with the calcium oxalate stone manifests itself in a significantly increased effective scatter coefficient 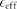. For an animated volumetric micro-CT rendering of both stones find Movie S1.

The classification of kidney stones by their scattering properties is more difficult, since the morphological structure and stone formation heavily depend on the mineralogical composition, the time varying chemical composition of the urine, the location and time of formation, the presence of growth inhibitors and catalyses, the inclusion of organic matrix, among others^19^,^20^. Thus, for the sake of simplicity, micro-CT investigations are restricted to pure uric acid and calcium oxalate stones in the following, signifying two micro-morphological extremes:

Uric acid type of kidney stones are known to grow in a layer-wise manner as concentric rings around a crystallite core^19^. This multi-shell structure is well reflected by the micro-CT measurement of an uricite stone as shown in Fig. 2A. The inner structure of the uricite stone displays a high textural irregularity comprising various grain sizes rutted with ring-like structures of higher optical density. Further, the exterior exhibits a distinct surface roughness containing large cavities and sharp edges. Since scatter predominately originates at boundaries with locally changing density and structure, uric acid stones are expected to have a large effective scatter coefficient 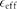.

In contrast, in the case of calcium oxalate stones crystal forming is typically driven by a slow and regular crystalline growth (the exact stone formation is complex and dependent on many factors, among others the presence of crystallization cores and the ratio of mono-hydrate to di-hydrate)^20^. As a consequence, especially in the case of calcium oxalate mono-hydrate, stones exhibit a relatively homogeneous micro-structure with wedges rounding off and forming a smooth exterior^21^. This corresponds well with the micro-CT measurements of a 90% mono-hydrate and 10% dihydrate stone featuring a strongly uniform micro-morphology with only minor structural disturbances in the form of some air cavities as shown in Fig. 2B. The high degree of regularity in grain size, a steady optical density throughout the stone and smooth stone surface manifest themselves in a very small effective scatter coefficient 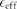.

Mixed types of stones comprise a more variable crystalline growth pattern, being more irregular in shape and structure than calcium oxalate stones, hence are expected to yield an intermediate effective scatter coefficient 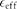.

### X-ray dark-field radiography of renal calculi

**Figure 3:**
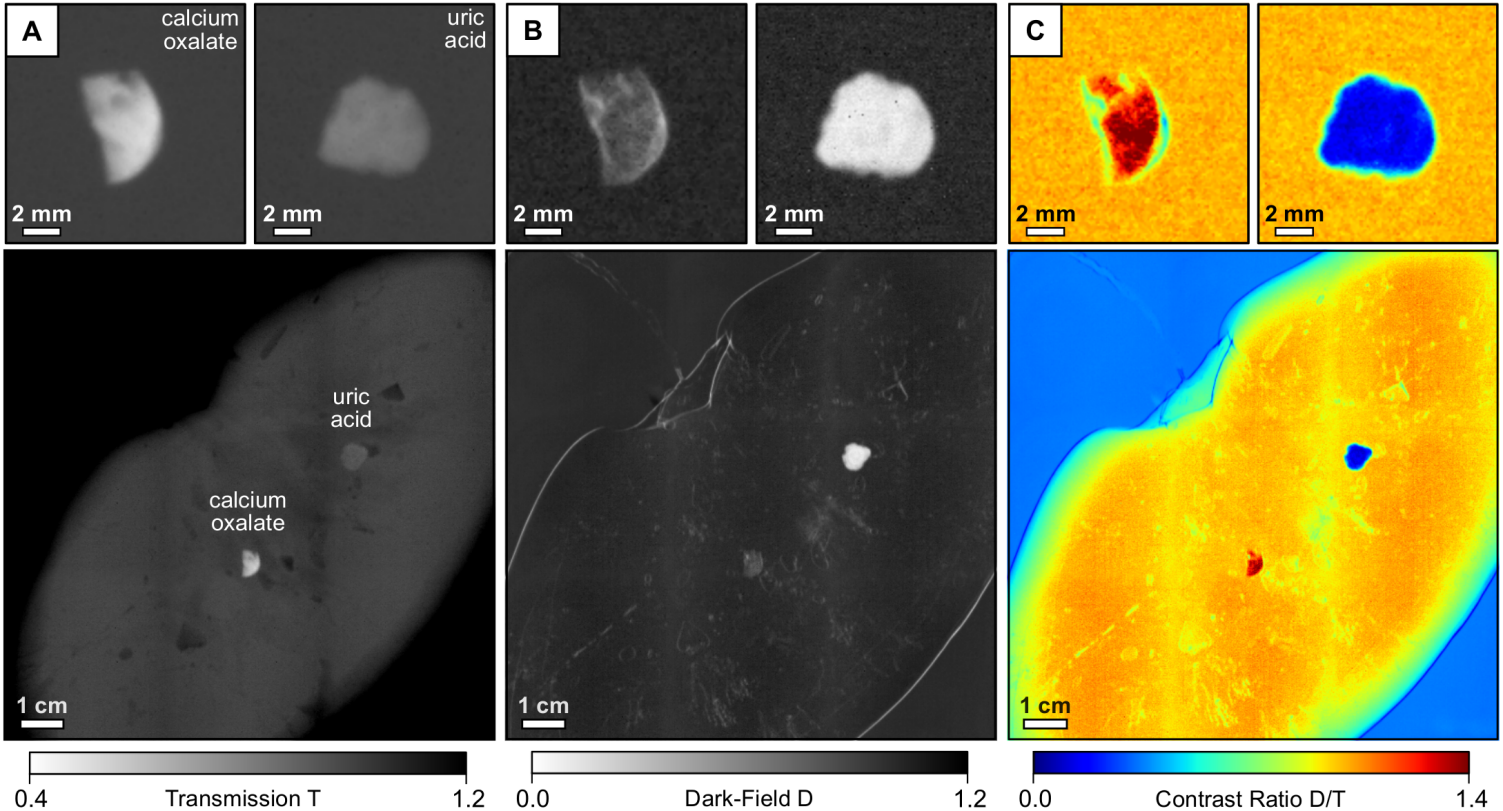
Visual classification scheme for the discrimination of uric acid and calcium oxalate renal stones using X-ray dark-field radiography. (A) Transmission images T of an calcium oxalate (top left), uric acid stone (top right) and a pig kidney with both stones embedded (bottom), taken at 40 kVp tube voltage. (B) Corresponding dark-field images D. (C) Since both stones show opposite absorption and scatter characteristics, the dark-field-to-transmission ratio D/T allows a simple visual differentiation of stone class and discrimination from surrounding kidney tissue in false color. Notice that the uric acid stone appears radio-lucent, while yielding high contrast in the dark-field image.

Grating-based transmission and dark-field radiographies of an excised calcium oxalate and uric acid stone can be seen in Fig. 3A & B. While the calcium oxalate stone (top left inlay) exhibits a relatively high absorption (low transmission T, large *μ*_eff_) and weak scattering signal (high dark-field D, small 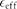), directly inverse observations are made in case of the uric acid stone (top right inlay), which is in accordance with previously discussed chemical and morphological stone properties. Based on the complementarity of both image signals, the two calculi can be clearly and easily differentiated in radiographic mode, as shown in Fig. 3C in false color, by deriving the thickness-independent dark-field-to-transmission ratio D/T. Afterwards, we measured a fresh pig kidney with both stones embedded in the inside with an imaging dose of 5.2 mGy (bottom inlays), to demonstrate the potential of kidney stone assessment via X-ray dark-field radiography as a future in-vivo application.

Consequently, a superior visual differentiation and discrimination between the two calculi and surrounding kidney tissue could be achieved also within native tissue. Notice that images were normalized with respect to surrounding tissue/material in order to compensate for signal not directly arising from the kidney stones themselves.

### Statistical analysis of renal calculi classification

**Figure 4:**
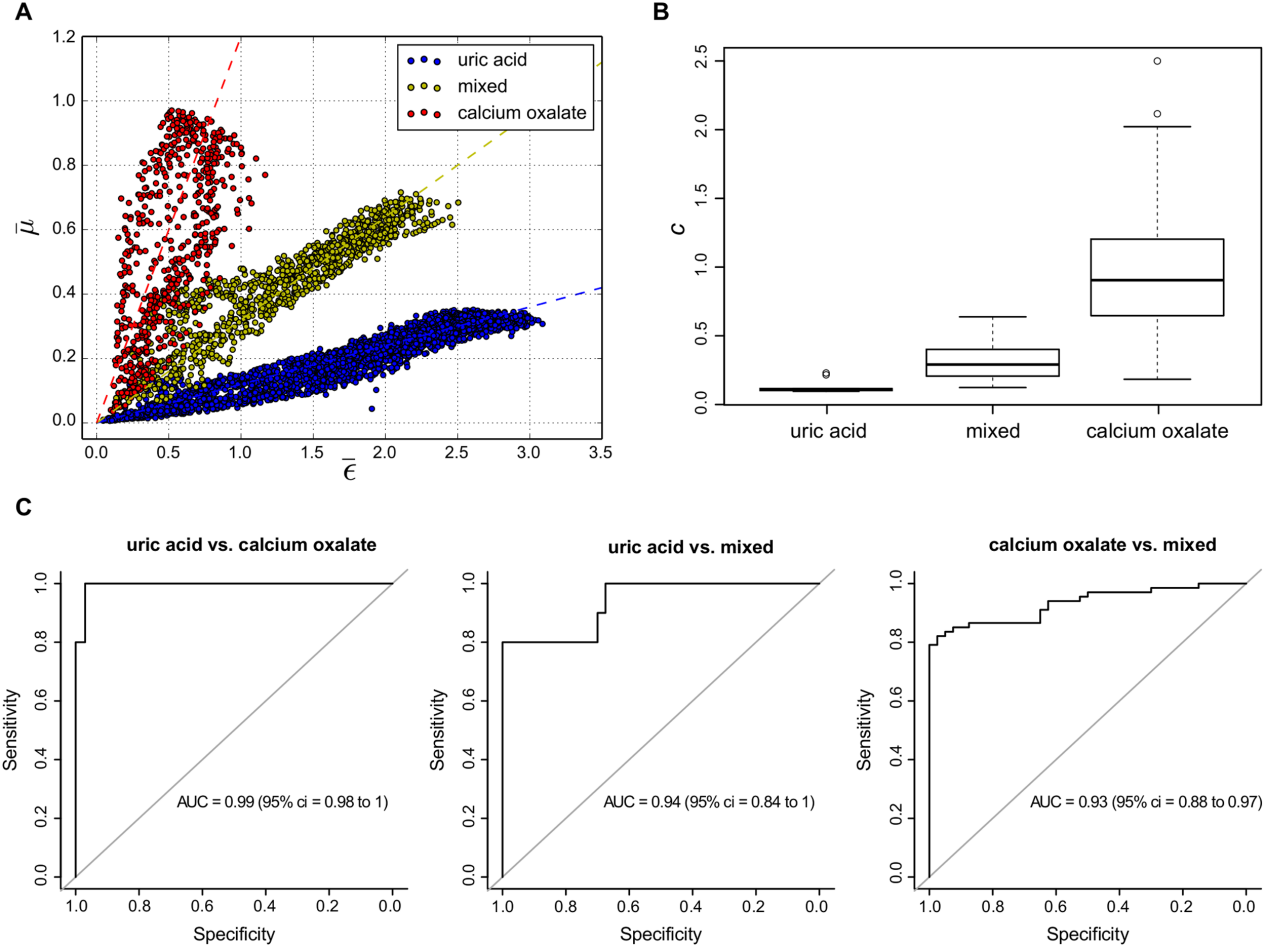
Statistical analyses adjudge superior diagnostic performance of kidney stones assessment via X-ray dark-field radiography. (A) Scatter plot showing the ratios of 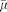 versus 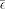 for every image pixel of an exemplary uric acid (blue), calcium oxalate (red) and mixed types of kidney stones (yellow). Each data-cloud was fitted by a linear regression as indicated by dashed lines, and its slope *c* used as a binary stone type classifier. (B) Box-whisker plot showing the distributions of slope values *c*, determined for the uric acid, calcium oxalate and mixed stone collective. In addition to strongly differing mean values (black line) no overlap in interquartile data was found (box). (C) Receiver operator characteristic analyses on the data presented in (B), show a highly sensitive and specific differentiation of all three stone classes using X-ray dark-field radiography (Area under curve > 0.9).

To evaluate this trend statistically, i.e. review the potential to differentiate uric acid and calcium oxalate from each other and also discriminate the latter from mixed stone types, a cohort of 118 stones was analysed. Each stone was segmented, normalized with respect to the background and separately analysed by generating a scatterplot of 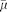 versus 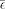 using every pixel of each stone as data-points, as exemplary shown for three stones in Fig. 4A. Here, data points close to the origin are associated with the margins of the respective stone, while the maximal values of 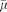 and 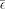 correspond to the region where the stone is vastest. Each point cloud belonging to a specific stone was then analysed separately using linear regression through the origin, evaluating the slope *c* (used as binary classifier) and the coefficient of determination *R*^2^ of each stone. For each class, the obtained slopes were then arithmetically averaged to obtain a mean slope value 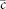 which is characteristic for each class. For uric acid stones, we obtain 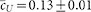, for the calcium oxalate 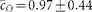, and for the mixed stones 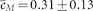. The error is obtained as the standard deviation of the slopes ensemble of each class. The coefficient of determination was used as an indicator of the goodness-of-fit and calculated to *R*^2^ = 0.80 ± 0.16 over the full sample collective, thus justifying the linear proportionality assumption expressed by Eq. 5.

We investigated the distributions of slope values *c* for each of the 118 stones in more detail by using a box-whisker diagram as shown in Fig. 4B. In addition to significantly differing median values (black dash), no overlap in interquartile data (50% of the data set as indicated by boxes) was observed for either of the three stone classes. Further, except two outliers (circles) all uric acid stones exhibited exclusively flatter slopes *c* than in comparison with the calcium oxalate stones.

### Diagnostic performance of renal calculi classification

To verify whether the decisive change in effective absorption 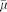 to scatter power 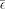 with respect to stone class yields sufficient diagnostic value, receiver operating characteristic (ROC) analyses were carried out. The ROC curve provides combinations of specificity vs. sensitivity when using the slope *c* as a binary classifier with varying threshold as shown in Fig. 4C. The diagnostic performance of renal calculi assessment via dark-field imaging was estimated by the area under the ROC curve (AUC). In the case of uric acid and calcium oxalate stones, a nearly unambiguous discernability between both classes was found using X-ray dark-field radiography, quantified by a AUC value of 0.99 (95% bootstrap confidence interval of 0.98 to 1). An optimal threshold value (Youden-Index), maximizing the sum of diagnostic sensitivity (100%) and specificity (97%), was found for *c* = 0.26. Besides, also mixed type of renal stones were found to be distinguishable with a high accuracy from both uric acid (AUC of 0.94, 95% bootstrap confidence interval of 0.84 to 1) and calcium oxalate stones (AUC of 0.93, 95% bootstrap confidence interval of 0.88 to 0.97).

## Discussion

Here, we have shown that the comparison of absorption and dark-field signal strength can determine the composition of different calculi classes of the genitourinary system. Our study was able to establish a clear trend in the absorption-to-scattering ratio, which we could directly assign to chemical and morphological differences of calcium oxalate and uric acid stones. We further deepened this correlation by means of statistical analyses and scatter plots. A simple visual inspection of the dark-field-to-transmission signal strength was presented to allow a quick and convenient determination of the stone type, compatible with clinical routine. Finally, receiver operator characteristics including 118 stones from 18 patients revealed an outstanding diagnostic performance of dark-field radiography for the accurate differentiation of pure uric acid and calcium oxalate calculi as well as discrimination of mixed types of stones. To secure our statistical findings and further clarify the origins of dark-field contrast with respect to stone micro-morphology, especially aiming at complicated, rare and mixed stone types, more work including an increased sample collective and patient cohort is to be performed in the near future. Follow-up studies will focus on the deduction of composite-specific classifier values, enabling a more detailed differentiation of mixed types of stones into their chemical sub-groups.

Besides, we consider X-ray dark-field radiography to provide a superior detection sensitivity towards certain renal stones in comparison to conventional radiography, due to the demonstrated complementarity of absorption- and scatter-based imaging. While uric acid stones are usually entirely radio-lucent, which involves a high risk of being overlooked in conventional radiography and CT (see Fig. 3A), they are clearly revealed and delineated by the dark-field signal (see Fig. 3B)^22^. Also in the case of mixed types of stones, the dark-field signal strength exceeds the respective absorption entity by far (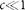, Fig. 4B), which is of major clinical interest taking into account that only 60% of all renal stones are radiopaque^23^.

As this initial study was aimed at determining the potential of dark-field imaging in the differentiation of kidney stones in the sense of a proof-of-principle study, mostly excised stones were measured within an ex-vivo framework. In a first step, we could successfully verify our classification scheme by fully scanning a fresh pig kidney with two manually embedded stones, while keeping the dose applied considerably low at 5.2 mGy. Although this value may not directly apply to a full abdomen scan, it is in the same order of magnitude as clinical dose values (0.7 mGy and 8.0 mGy in case of an abdomen radiogram and CT, respectively)^24^. Thus we are convinced that dose-compatible abdomen dark-field radiography could be achieved, considering that an optimization of interferometer efficiency by tuning several setup entities (grating height and quality, duty cycle, beam energy and filtration) would imply a significant decrease in dose, while maintaining equivalent image quality (for a more detailed dose discussion, see Refs. 25, 26).

**Figure 5:**
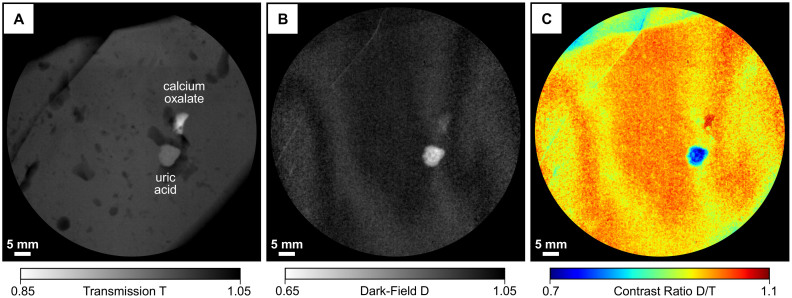
In-Vivo transferability study at 100 kVp tube voltage using a preliminary abdomen phantom. (A) High-energy transmission image T of a pig kidney, with manually embedded uric acid and calcium oxalate stones, placed within a 11 cm water-bath. (B) Corresponding dark-field image D, revealing concentric growth rings of the uric acid stone. (C) The dark-field-to-transmission D/T signal enables a clear differentiation of uric acid (blue) and calcium oxalate (red), consistent with the 40 kVp measurements shown in Fig. 3.

For the purpose of further pursuing clinical transferability of the proposed method in the near-term, we modelled a first medical meaningful scenario mimicking an abdomen phantom: A second pig kidney, with both stones embedded was placed within a 11 cm water-bath and measured at a high-energy laboratory setup running at 100 kVp, which is the energy range of tube voltages used in commercial systems. The preliminary obtained measurements shown in Fig. 5 proves that an inversion of dark-field-to-transmission signal when comparing uric acid and calcium oxalate stones is still existent, even in the case of very high X-ray energies (*E*) and significant beam-hardening. Future studies have to investigate, whether this does hold true for the differentiation from mixed types of stone, considering that *μ*_eff_ and 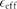 are proportional to *E*^−3^ and *E*^−2^, respectively^15^. An increase in mean energy namely implies the drawback of converging and shrinking scatter plots offering a correspondingly reduced diagnostic performance.

Further an animal model is envisioned to address concerns regarding the impact of structures overlying the kidney in radiographic imaging mode and discriminability of small and initially growing calculi in-vivo^27^. Especially in the case of inhomogeneous structure compositions or strongly absorbing tissue underlying the kidney stone data normalization could be ambiguous, resulting in a limited accuracy in the determination of the stone type classifier *c*. Finally, X-ray dark-field radiography is susceptible to superposing renal stones; hence an additional lateral radiogram of the abdomen or the implementation of advanced techniques such as tomosynthesis would be necessary to support diagnostic reliability. Besides, current technical limitations need to be challenged, such as the fabrication of bended, large-field-of-view gratings with high aspect ratios, in order to significantly reduce scan-time and secure a successful implementation of non-invasive kidney stone assessment via X-ray dark-field radiography into clinical routine.

## Methods

### Study design

A broad range of different kidney stones comprising various stone sizes was acquired in order to have a representative sample collective. Nevertheless, the classification of kidney stones is in general complicated by the fact that most kidney stones in practice are rarely composed of a single pure chemical material, but are instead a mixture of various components with widely differing composition^28^. Thus in this proof-of-principle study we focus on the differentiation of three classes of kidney stones only: the pure uric acid stones, the pure calcium oxalate stones, and the mixed stone class including composites of brushite, carbon apatite and struvite. The different kidney stone types that occur in practice are summarized in table 1 by compound name, chemical formula and mineralogical name.

The samples in our measurements were acquired by the Klinikum rechts der Isar, Department of Radiology. Each patient had their renal stone(s) removed following the common clinical practice with respect to their individual diagnosis and indication. Written and informed consent was obtained from all patients. Nine patients were found with a mixture of Whewellite and Weddelite, four patients with uric acid stones, and five patients with mixed stones types. From these patients, we obtained 68 oxalate stones, 10 uric acid stones, and 40 mixed stones, thus a total of 118 renal calculi was accessed and imaged.

The composition of each kidney stone was determined by Fourier-transform infrared spectroscopy (FTIR)^29^, using a Spectrum 100 system by Perkin Elmer, Beaconsfield, UK. The exact chemical composition of the calculus was determined by comparing the recorded spectrum with tabulated spectra. Components of stones were given in percentages, in which a concentration of more than 90% of one component was regarded as pure. More details on the study design, patient selection routine, patient examination and exact composition of the kidney stone collective can be found in Ref. 5.

### Statistical analysis

Statistical analysis was carried out using the statistical software *R* and its library *pROC*^30^,^31^. Outliers were considered in the ROC analysis. Two thousand stratified bootstrap samples were drawn for each ROC analysis to estimate 95% confidence intervals for the area under the ROC curve.

### Micro-CT setup

We performed micro-CT measurements at a commercial GE VtomeX system, using a reflection tube with a voltage setting of 100 kVp at a current of 10 mA and a voxel-size of 20 × 20 × 20 μm^3^. For the tomographic scan we took 1200 projections over 360 degrees, with an exposure time of 1 s each. Datasets were reconstructed using a standard filtered backprojection, rendered in Volume Graphics VGStudio MAX and analyzed visually. An example volume rendering can be seen in Fig. 2.

### X-ray dark-field interferometer

X-ray dark-field radiography was conducted with a compact laboratory setup using a three-grating Talbot-Lau interferometer (effective pixel-size of 85 × 85 μm^2^)^9^. The source is a Nonius FR 591 rotating anode tube with molybdenum target, operated at 40 kVp and 70 mA. The beam-splitter grating *G*_1_ is a *π*/2-shifting binary phase-grating with a design energy of 27 keV. The interferometer is built in an asymmetric geometry with periods of 10 μm, 4.8 μm and 3.24 μm for *G*_0_, *G*_1_ and *G*_2_, respectively. The setup length is 1570 mm, with inter-grating distances of *l* = 1060 mm between *G*_0_ and *G*_1_ and *d* = 510 mm between *G*_1_ and *G*_2_, corresponding to the third Talbot order at the design energy. The samples are positioned 80 mm downstream of *G*_1_. The contributions of absorption and scattering in the projections are separated using a phase-stepping technique^8^, with 14 phase-steps and 4 seconds exposure time each. Transmission and dark-field signals are then obtained from the raw phase-stepping projection data using a Fourier analysis approach. Exemplary transmission and dark-field projections can be seen in Fig. 3. For the statistical analysis, we normalized the projections with respect to the sample holder, in order to remove all contributions not originating from the kidney stone. All stones were then segmented from the background by intensity thresholding and their 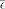 and 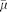-values were tabulated for each single pixel for further analysis. For the purpose of validating in-vivo feasibility of renal calculi assessment via dark-field radiography, one calcium oxalate and one uric acid stone were additionally embedded and subsequently measured within a fresh pig kidney (Fig. S1). We derived comprehensive images of the pig kidney with a field-of-view of 12.8 × 12.8 cm^2^ by stitching 16 single projections taken with 5 phase-steps and 1 second exposure time each (for a more detailed description, see Reference 25). Each projection was scanned within 20 seconds (overall scan time of 320 seconds).

### Imaging dose

The total air kerma of the pig kidney measurement was determined to be 10.5 mGy (incident air kerma rate 2.1 mGy/s) with a Dosimax plus/RQX-detector system. To give a rough estimate of the effective dose deposited in the kidney, we calculated the mean glandular dose of a 100% glandular breast tissue equivalent (underlying consideration are based on the fact that glandular breast and kidney tissue yield similar mass density coefficients)^32^. We obtained a mean dose of 5.2 mGy, by multiplying the total incident air kerma with a Monte-Carlo based conversion factor of 0.56 and a correction factor accounting for 100% glandular breast tissue of 0.9. Values arise from a half-value layer (Al) of 0.8 mm and a kidney thickness of 3 cm^33^,^34^.

### Potential clinical implementation

In a first step, the radiologist marks the renal stone in the digital radiogram. Since stone edges yield excessive scatter corresponding regions are excluded from the data analysis. Afterwards the region surrounding the kidney stone is automatically selected in the radiographs and the respective mean signal values in both the absorption and dark-field channel calculated, by which the renal stone is subsequently normalized. This is feasible since the kidney stone is distinctively thinner than the patient. In a next step, the scatter plot is generated from 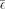 and 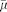 and the binary stone classifier *c* extracted as the mean slope of the data points, by which the respective stone type can be determined from a look-up table. Finally, by reading the maximum ordinate of the scatter plot (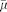) the thickness of kidney stone in beam direction can be approximated.

## Author Contributions

K.S., E.B., K.W. and M.W. performed the measurements. K.S., P.N., E.R. and F.P. conceived the study. M.E., A.F., M.S. and H.S. provided and analyzed the sample collective. B.H. performed the statistical analysis. K.S., M.C., J.H. and A.F. wrote the manuscript.

## Supplementary Material

Supplementary Information Supplementary Movie S1

Supplementary Information Supplementary Information

## Figures and Tables

**Figure 1:**
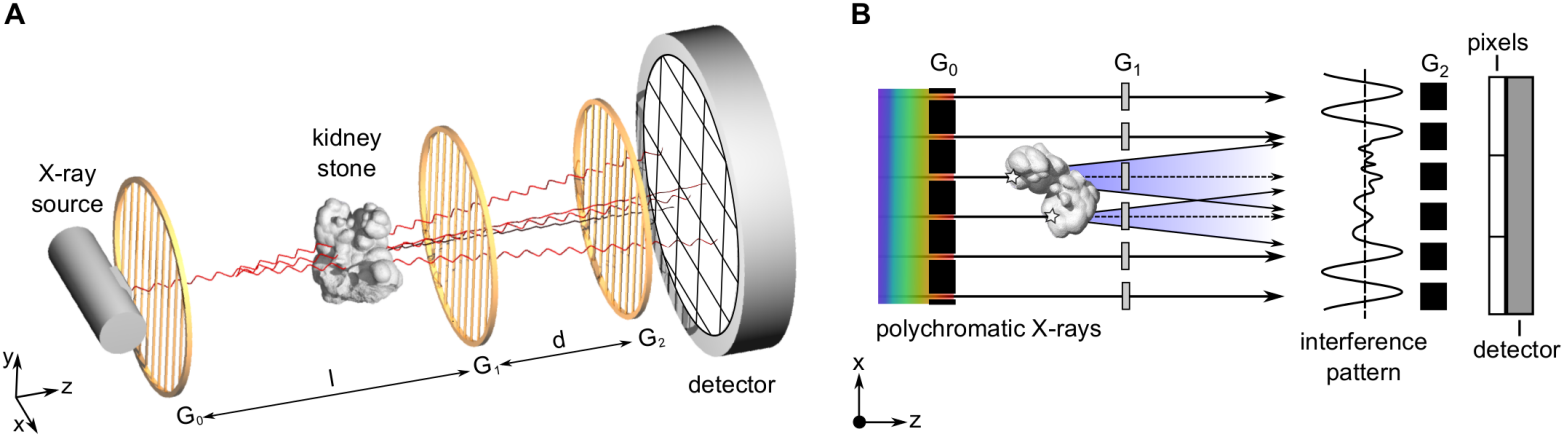
Contrast generation in grating-based X-ray dark-field imaging. (A) Typical setup with a conventional X-ray source, an source grating G_0_, a phase grating G_1_, an analyzer grating G_2_ and a flat panel detector. (B) Diffuse X-ray scatter originating from sub-structures of the kidney stone manifest themselves in a local reduction of a phase-grating induced interference pattern. By analysing the dark-field signal, information on the kidney stone micro-morphology, well below the detector resolution limit can be retrieved.

**Table 1:** Overview of the different types of kidney stones relevant for this study, with chemical name, formula and mineralogical name. Stoichiometry adapted from Ref. 28

compound name	chemical formula	mineralogical name
Calciumoxalate-Monohydrate	*CaC*_2_*O*_4_ · *H*_2_*O*	Whewellite
Calciumoxalate-Dihydrate	*CaC*_2_*O*_4_ · 2*H*_2_*O*	Weddelite
Uric acid	*C*_5_*H*_4_*N*_4_*O*_3_	Uricite
Uric acid dihydrate	*C*_5_*H*_4_*N*_4_*O*_3_ · 2*H*_2_*O*	none
Ammonium urate	(*NH*_4_)*C*_5_*H*_3_*N*_4_*O*_3_	none
Sodium urate monohydrate	*NaC*_5_*H*_3_*N*_4_*O*_3_ · *H*_2_*O*	none
Calcium hydrogenate phosphate	*CaH*(*PO*_4_) · 2*H*_2_*O*	Brushite
Carbonate hydroxylapatite	*Ca*_5_(*PO*_4_, *CO*_3_)_3_(*OH*)	Apatite/Dahllite
Magnesium ammonium phosphate	*Mg*(*NH*_4_)(*PO*_4_) · 6*H*_2_*O*	Struvite
Cystine	*C*_6_*H*_12_*N*_2_*O*_4_*S*_2_	none
