# Supplementary material for: Non-invasive Differentiation of Kidney Stone Types using X-ray Dark-Field Radiography
Source: Sci Rep. 2015 Apr 15;5:9527. doi: 10.1038/srep09527 (PMC4397641; doi:10.1038/srep09527)
Supplement: Supplementary Information [file srep09527-s2.pdf]

Non-invasive Differentiation of Kidney Stone Types using X-ray Dark-Field Radiography

Kai Scherer[1], Eva Braig[1], Konstantin Willer[1], Marian Willner[1], Alexander Fingerle[2], Michael Chabior[1], Julia Herzen[1], Matthias Eiber[2], Bernhard Haller[3], Michael Straub[4], Heike Schneider[5], Ernst Rummeny[2], Peter Noël[2], Franz Pfeiffer[1]

[1]Department of Physics and Institute of Medical Engineering, Technische Universität München, Garching, Germany

[2]Department of Radiology, Technische Universität München, Munich, Germany

[3]Department of Medical Statistics and Epidemiology, Technische Universität München, Munich, Germany

[4]Department of Urology, Technische Universität München, Munich, Germany

[5]Department of Clinical Chemistry and Pathobiochemistry, Technische Universität München, Munich, Germany

Correspondence: Kai Scherer, Department of Physics and Institute of Medical Engineering, Technische Universität München, James-Frank-Strasse 1, Garching, Germany, Phone 49.89.289.12552, Fax 49.89.289.12548

E-mail: kai.scherer@tum.de

1. Supplementary Materials

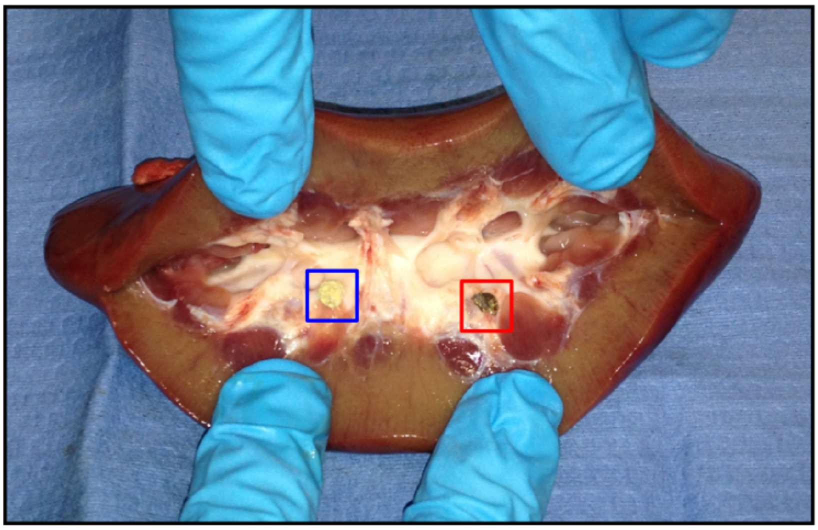

Fig. 1 Photography of a fresh pig kidney with renal stones embedded, used to show-cast X-ray dark-field radiography. One exemplary uric acid (blue framed) and one calcium oxalate stone (red framed) were manually embedded in the fresh pig kidney before measurement.

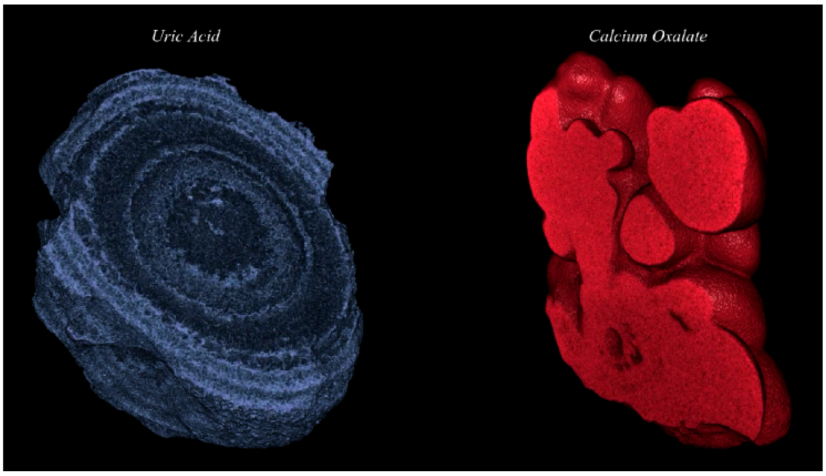

Fig. 2 Volumetric micro-CT rendering of an exemplary uric acid and calcium oxalate stone - Preview. Significant differences in the micro-morphology of uric acid and calcium oxalate stones determine the dark-field signal strength of the respective stone type.
